# Amplicon deep sequencing improves *Plasmodium falciparum* genotyping in clinical trials of antimalarial drugs

**DOI:** 10.1038/s41598-019-54203-0

**Published:** 2019-11-28

**Authors:** Maria Gruenberg, Anita Lerch, Hans-Peter Beck, Ingrid Felger

**Affiliations:** 1 Swiss Tropical and Public Health Institute, Basel, Switzerland; 2 University of Basel, Basel, Switzerland; 3 Present Address: Eck Institute for Global Health, University of Notre Dame, Notre Dame, USA

**Keywords:** Malaria, Epidemiology, Outcomes research

## Abstract

Clinical trials monitoring malaria drug resistance require genotyping of recurrent *Plasmodium falciparum* parasites to distinguish between treatment failure and new infection occurring during the trial follow up period. Because trial participants usually harbour multi-clonal *P. falciparum* infections, deep amplicon sequencing (AmpSeq) was employed to improve sensitivity and reliability of minority clone detection. Paired samples from 32 drug trial participants were Illumina deep-sequenced for five molecular markers. Reads were analysed by custom-made software HaplotypR and trial outcomes compared to results from the previous standard genotyping method based on length-polymorphic markers. Diversity of AmpSeq markers in pre-treatment samples was comparable or higher than length-polymorphic markers. AmpSeq was highly reproducible with consistent quantification of co-infecting parasite clones within a host. Outcomes of the three best-performing markers, *cpmp, cpp* and *ama1-D3*, agreed in 26/32 (81%) of patients. Discordance between the three markers performed per sample was much lower by AmpSeq (six patients) compared to length-polymorphic markers (eleven patients). Using AmpSeq for discrimination of recrudescence and new infection in antimalarial drug trials provides highly reproducible and robust characterization of clone dynamics during trial follow-up. AmpSeq overcomes limitations inherent to length-polymorphic markers. Regulatory clinical trials of antimalarial drugs will greatly benefit from this unbiased typing method.

## Introduction

The efficacy of new drugs against *Plasmodium falciparum* is tested *in vivo* in clinical trials conducted in malaria endemic field sites, where transmission is ongoing. Therefore, trial participants may be exposed to infection by new parasite clones during the trial period. New infections (NI) coming in during follow-up can severely compromise trial outcomes because detection of recurrent parasitemia in participants is scored as drug failure, despite initial successful parasite clearance by the drug under trial. Drug trials conducted in moderate and high transmission settings thus suffer from underestimation of therapeutic efficacies. Differentiation of true treatment failures from new infections is therefore crucial for efficacy trials as well as for drug resistance surveillance in the field.

The problem deriving from parasite clones acquired during the follow-up period was resolved by genotyping parasites at the day of treatment and at the day of recurrent parasitemia. Highly diverse length-polymorphic antigen markers or microsatellites had been used for this purpose and laboratory and analytical procedures have been harmonized at a stakeholder meeting organized by WHO and MMV in 2007^1^. In the past years, we and others observed that the established length-polymorphic markers, i.e. genes for merozoite surface protein 1 and 2 (*msp1, msp2*) and glutamate-rich protein (*glurp)*, likely are suboptimal, as major size differences between amplicons cause a bias in amplification efficiency, which in multi-clonal infections leads to preferred amplification of shorter fragments and loss of long alleles^2–10^. Therefore, the development of alternative, simple, high-throughput, and highly sensitive genotyping techniques is particularly important for discrimination of recrudescent from new infections in regulatory trials.

We hypothesized that marker genes rich in single nucleotide polymorphism (SNP) may be superior to the standard length-polymorphic markers that contain intragenic tandem repeats. SNP-rich marker genes are genotyped by deep sequencing of PCR amplicons (AmpSeq)^11–15^. Amplification bias, as observed with length-polymorphic markers, should be overcome by choosing amplicons of the same length. The question on how the established *msp1/msp2/glurp* genotyping method compares with AmpSeq regarding marker diversity, multiplicity of infection (MOI), and trial outcomes, was addressed by applying both typing techniques to the same set of samples.

This study investigated the utility and benefits of amplicon deep sequencing applied for genotyping of *P. falciparum* in a clinical trial of antimalarial drug efficacy. Development, validation and down-selection of molecular markers, together with an improved protocol and data analyses pipeline, aimed at a robust and reproducible new genotyping technique for PCR-correction of treatment failure rates that is acceptable by regulatory authorities.

## Results

Using data from the MalariaGen genome data repository^16^ we identified highly diverse molecular markers that show extensive single nucleotide polymorphism (SNP) according to bioinformatics strategies described earlier^15^. Here, two new *P. falciparum* AmpSeq markers, *cpp* and *msp7*, were developed and validated, which were used together with 3 previously identified AmpSeq markers^14,15,17^ *ama1-D3, cpmp*, and *csp*, for differentiation of NI and R by deep amplicon sequencing in paired samples from a clinical trial of an antimalarial drug.

### Analysis of Illumina sequence reads

Starting from DBS, we amplified and sequenced 34 paired pre- and post-treatment field samples and 6 control mixtures of *P. falciparum in vitro* strains HB3 and 3D7 for the five markers *ama1-D3*, *cpp*, *cpmp*, *csp* and *msp7* using an Illumina MiSeq platform in paired end mode. Sequence reads were processed with software HaplotypR resulting in a median coverage of 13698 [range 2826–21853, 2.5% and 97.5 percentile] per sample and marker. Median sequence coverage per amplicon over all sequenced samples agreed well between pre- and post-treatment samples (Supplementary file, Table S1). Two paired samples had to be excluded from the PCR-correction analysis because of poor DNA quality and limited DBS starting material for two of the post-treatment samples.

### Definition of cut-off settings

**Figure 1:**
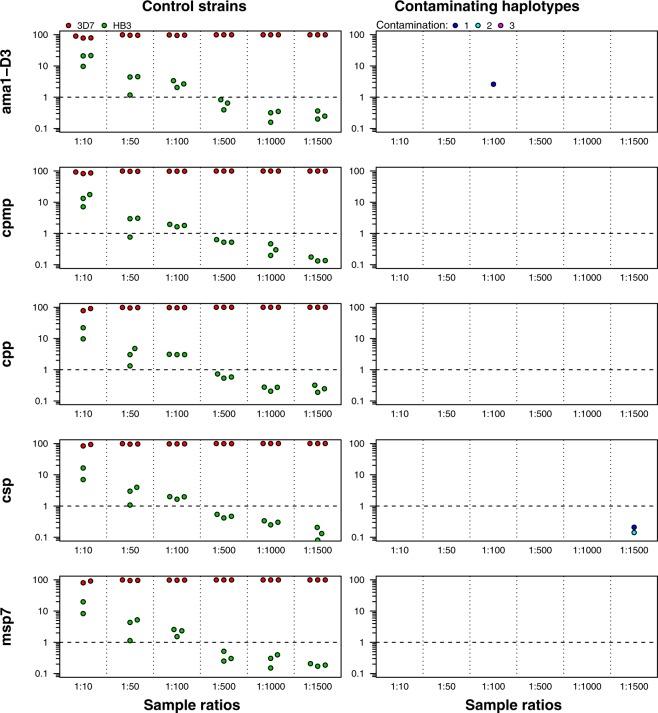
Haplotypes detected in experimental mixtures of *P. falciparum* strains HB3 and 3D7. Proportion of *ama1-D3, cpmp, cpp, csp*, and *msp7* haplotype reads from mixtures of two *P. falciparum in vitro* culture strains HB3 (green) and 3D7 (red) (left panel) and detection of rarely observed, contaminating haplotypes (right panel). HB3 / 3D7 ratios 1:10 to 1:1500. Y-axis: proportion of reads. Dashed horizontal lines: cut-off for minority clones set at 1% of total reads. No data was available for one of the replicates of markers *cpp*, *csp* and *msp7* of ratio 1:10.

The most crucial part in generating robust genotyping results from deep sequencing of amplicons was the definition of cut-off settings. On the one hand, a stringent cut-off is required for excluding sequencing errors; on the other hand, a less stringent cut-off would be desirable for maximized detection of minority clones. In view of the robustness and high reproducibility required for genotyping in regulatory clinical trials, we aimed for a conservative choice of cut-off setting that would rather overestimate than underestimate treatment failure rates. For a rational decision on cut-off settings for the data analysis pipeline, we have genotyped *P. falciparum in vitro* culture strains HB3 and 3D7 mixed in different ratios (Fig. 1).

Controlled conditions with known sequences and defined mixing ratios permitted (i) a thorough validation of cut-off settings for stringent haplotype calling and (ii) an evaluation of minority clone detectability at different cut-off settings. Applying cut-off settings of a minimum of 10 reads/haplotype and 50 reads/sample, all five AmpSeq markers identified the original haplotypes HB3 and 3D7 correctly at six different ratios (Fig. 1). The minority clone was detected correctly up to the highest dilution of 1:1500. However, applying the chosen default cut-off of 1% censored minority clones in all dilutions exceeding a ratio of 1:100. Minority clones are robustly detected up to a dilution ratio of 1:100 when applying these default cut-off settings.

Reproducibility of results was assessed by analysing independent triplicates from HB3/3D7 control mixtures generated by the same AmpSeq protocol as used for field samples. Comparing haplotypes in all three replicates permitted detection of false haplotype calls that might originate from either PCR artefacts or cross- sample or environmental DNA contamination and occur in one of the replicates. False *ama1-D3* and *csp* haplotypes were found in a single replicate of one control sample (Fig. 1, right panel).

The false *csp* haplotype calls fell below 1% within-host frequency cut-off. We found that erroneous haplotypes occurred only in 1/3 replicates and were rarely observed in the HB3/3D7 control samples. Resulting true haplotypes were called with a minimum occurrence in at least two of the replicates to prevent erroneous haplotype calling resulting from rare PCR contamination. These control experiments highlighted, that performing AmpSeq genotyping for each sample in triplicate was essential for a reliable exclusion of erroneous haplotypes.

### Haplotype diversity of 5 AmpSeq markers in baseline samples

The genetic diversity of 5 molecular markers was assessed in 34 baseline samples with regards to number of SNPs observed per amplicon, number of haplotypes, mean multiplicity of infection (MOI) and expected heterozygocity, H_e_. In our baseline samples, marker *cpmp* showed highest discriminatory power with 63 haplotypes followed by markers *cpp* and *ama1-D3* (51 and 43 haplotypes) (Table 1). Less diversity was detected for markers *csp* and *msp7* (29 and 24 haplotypes). In agreement with the original WHO/MMV recommended genotyping approach, the three best performing markers, *cpmp, cpp*, and *ama1-D3*, were selected based on their H_e_ values as genotyping markers for the present study.

**Table 1:** Haplotype diversity of selected AmpSeq markers in 34 pre-treatment samples.

Marker	Size (bp)	Number of SNPs	Haplotypes	H_e_	MOI
*cpmp*	394	56	63	1.00	2.67
**PF3D7_0104100**					
*cpp*	362	29	51	1.00	2.67
**PF3D7_1475800**					
*ama1-D3*	493	16	43	0.98	2.51
**PF3D7_1133400**					
*csp*	298	16	29	0.97	2.63
**PF3D7_0304600**					
*msp7*	343	25	24	0.91	2.15
**PF3D7_1335100**					

**Figure 2:**
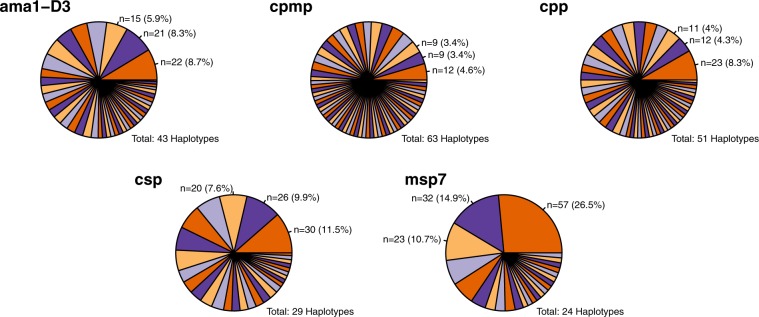
Haplotype frequencies for five AmpSeq markers in 34 pre-treatment samples.

Marker *msp7* showed 3 dominant haplotypes accounting for 50% of all *msp7* sequence reads (Fig. 2). The likelihood that two unrelated parasites share by chance the same haplotype is therefore greater for marker *msp7* compared to other markers. This chance effect could lead to underestimation of MOI and misclassification of NI as R by marker *msp7*. Consequently, we excluded marker *msp7* from the analysis of the present set of paired samples. Yet, marker *msp7* may well prove useful for other study sites, where this marker may have a greater discriminatory power. Similarly, marker *csp* showed slightly smaller diversity in baseline samples, where one predominant allele was observed with a frequency of 11.5% (Fig. 2). In case of missing data by any of the three prioritized markers, the result of backup marker *csp* was utilized.

**Figure 3:**
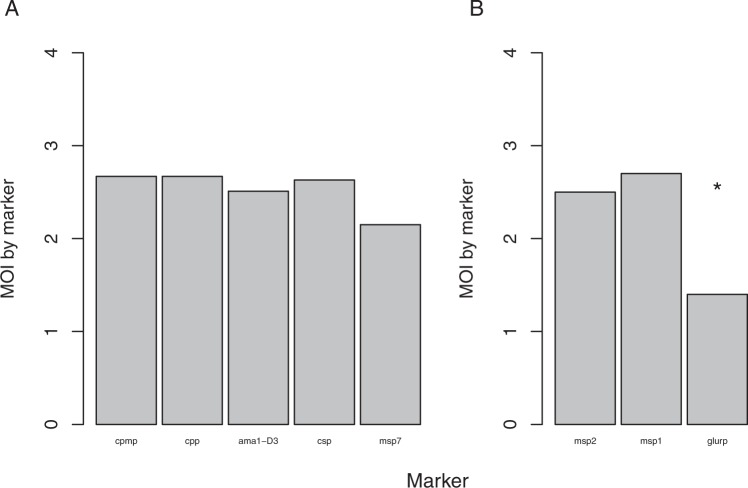
Mean multiplicity of infection (MOI) in 34 pre-treatment samples. MOI determined in 34 pre-treatment samples by five AmpSeq markers (**A**) and three length-polymorphic markers (**B**; data published previously^8^) (*p < 0.05 Wilcoxon test).

Mean multiplicity of infection (MOI) determined by all 5 AmpSeq markers in baseline samples was compared to MOI of the established length-polymorphic genotyping marker *msp2*, which had been reported for the same samples previously^8^ (Fig. 3). AmpSeq-based mean MOI was highest for marker *cpmp* (MOI = 2.67) and lowest for marker *msp7* (MOI = 2.15), likely owing to the occurrence of three dominant *msp7* haplotypes. We observed good concordance in mean MOI among the three prioritized AmpSeq markers as well as with the established and widely used length-polymorphic markers *msp1* and *msp2* (Fig. 3). Diversity of marker *glurp* is high in sequencing data repositories, but strong amplification bias during PCR obviously led to a significantly lower *glurp* MOI compared to MOI by marker *msp1* and *msp2* (p < 0.05 Wilcoxon test). Such a loss of the longer *glurp* fragments through size-mediated amplification bias in multi-clone infections has been described previously^8^.

### AmpSeq genotyping of paired pre- and post-treatment samples

A total of 34 archived paired pre- and post-treatment samples from *in vivo* clinical drug trials were genotyped for PCR-correction using AmpSeq. Markers *ama1-D3*, *cpmp*, and *cpp* with the highest discriminatory power were chosen for genotyping. In case a marker failed to amplify, *csp* was used to complement a full set of three individual markers. In three post-treatment samples, marker *cpmp* failed to achieve sufficient sequencing depth and marker *csp* was used instead.

**Figure 4:**
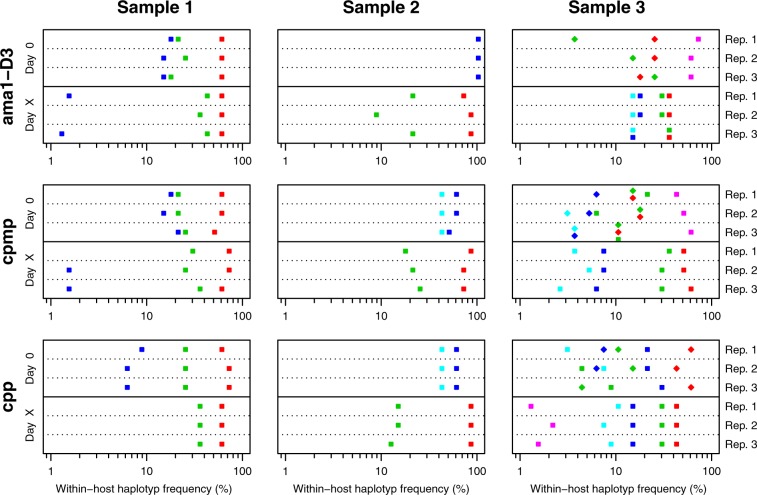
PCR-correction outcomes by AmpSeq for three sample pairs from Day 0/Day X. Within-host haplotype frequencies are shown for AmpSeq markers *ama1-D3, cpmp*, and *cpp*, quantified in three replicates (Rep1 to Rep3) for each of the Day 0 and Day X samples. Different haplotypes of a marker are represented by different shapes and colours. Left panel represents a clear recrudescence because of concordant results by all 3 markers; middle panel: clear new infection; right panel: discordant results whereby result from marker *ama1-D3* (NI) contrasts with that from *cpmp* (R) and *cpp* (R).

**Figure 5:**
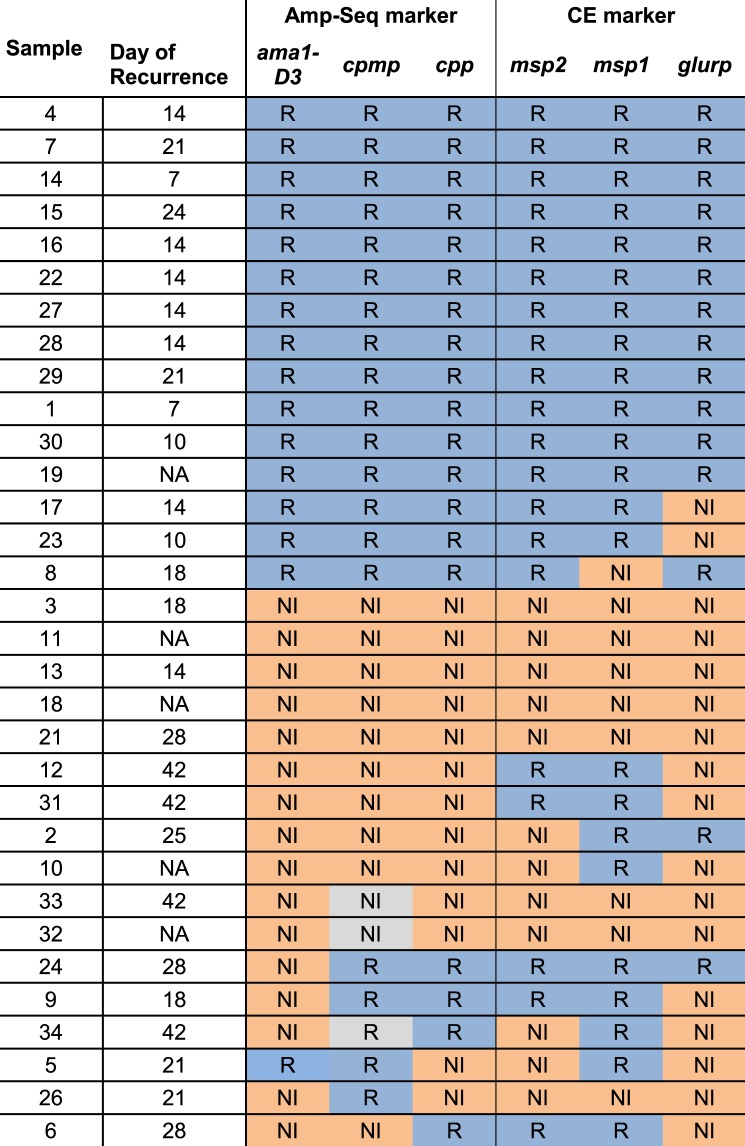
Genotyping results by three molecular markers for 32 paired pre- and post-treatment samples. Individual marker results for *ama1-D3*, *cpmp* and *cpp* by AmpSeq (left panel) and length-polymorphic markers *msp2*, *msp1* and *glurp* (right panel) are shown. Blue cell: Recrudescence. Orange cell: New Infection. Grey cell: Result of marker *csp* was used for 3 samples where marker *cpmp* failed to amplify.

Pre- and post-treatments samples were run in triplicates to prevent any overestimation of haplotypes resulting from PCR artefacts or PCR contamination. A haplotype needed to occur in at least two out of three independent replicates to be considered as a true haplotype. In a first step, the results of individual markers were classified. A sample pair was classified as R, if one or more haplotypes occurred in at least two replicates of both, the pre- and post-treatment samples, at a within-host haplotype frequency of ≥1%. A NI was defined by the presence of only new haplotypes in the post-treatment sample. Figure 4 illustrates an example for each of three possible genotyping outcomes: (i) a clear R with concordant results by all three markers (left panel), (ii) a clear NI (middle panel), and (iii) a discordant outcome where one marker disagreed with results of the other two markers (right panel). Haplotype calls of markers *ama1-D3, cpmp*, and *cpp* is shown at day 0 (pre-treatment sample) and day X (day of recurrent parasitemia). Additional figures for all remaining samples are provided in Supplementary file, Fig. S1 and S2. The individual genotyping results derived from markers *ama1-D3*, *cpp*, *cpmp* (or *csp*, in case of missing *cpmp* data in three samples*)* agreed in 81% (26/32) of sample pairs. AmpSeq identified 15 clear recrudescences and 11 clear new infections (Fig. 5, left panel). Discordant results among the three markers were seen in six sample pairs.

### Comparison of PCR-correction results by AmpSeq versus fragment-length polymorphic markers

The 32 sample pairs analyzed by AmpSeq had been genotyped previously using length-polymorphic markers *msp1, msp2*, and *glurp*^8^. These earlier results were used in this study for a direct comparison of the two genotyping methods (Fig. 5, right panel). Eight clear NI were observed plus 13 clear R, whereas the remaining 11 pairs presented different results in one marker and thus were classified as discordant. For length-polymorphism based genotyping, more sample pairs were discordant among the three markers than for AmpSeq genotyping. *Msp1, msp2*, and *glurp* genotyping results were concordant for 21/32 pairs, whereas by AmpSeq genotyping, 26/32 pairs gave concordant results (Fig. 5). Thus, the proportion of discordant results was substantially reduced from 34% (11/32) by length-polymorphic markers to 19% (6/32) by AmpSeq markers.

**Figure 6:**
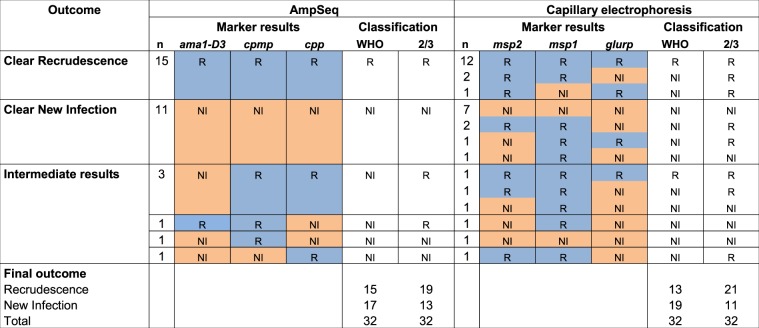
Comparison of PCR-correction outcomes determined by AmpSeq and length-polymorphic genotyping for 32 paired pre- and post-treatment samples. PCR-correction outcomes were generated by AmpSeq markers (left panel) versus length-polymorphic markers sized by capillary electrophoresis (right panel; published data^8^). For 3/32 sample pairs marker *cpmp* failed to amplify and was replaced by marker *csp*). For resolving discordant results between the three markers, both the WHO and the “two out of three markers” (2/3) algorithms were applied.

In a second step, the final PCR correction outcome for sample pairs with discordant results among the three markers was determined. The six discordant AmpSeq results were resolved using two alternative algorithms, the WHO recommended approach^1^ and the 2/3 algorithm^8^. Following the WHO approach, all six discordances would be classified as NI, because at least one of three markers indicated NI (Fig. 6, left panel). In contrast, by the 2/3 algorithm, the matching result of two markers will determine the final outcome. Accordingly, two of six discordant samples by AmpSeq were classified as NI, because two markers indicated NI, whereas the remaining four discordances showed R in two markers and were thus classified R in the overall result.

Also for length-polymorphic markers, the final PCR correction outcome was determined by both algorithms, WHO and 2/3 (Fig. 6, right panel; data published previously^8^). By WHO algorithm, all 11 discrepant samples would be classified as NI but according to the 2/3 algorithm, eight of these would be classified R. Thus, when using length-polymorphic markers for genotyping, the algorithm selected had a profound effect on treatment failure rates, with eight paired samples classified differentially. This effect was much less pronounced with AmpSeq, where only three paired samples showed different final outcomes when using the different algorithms. The smaller number of differences observed with AmpSeq implies that more robust results are generated. Using the 2/3 algorithm, both genotyping techniques resulted in similar numbers of treatment failures, with 59.4% (19/32) by AmpSeq and 65.6% (21/32) by length-polymorphic markers. This difference between final outcomes was not statistically significant (McNemars test; p = 0.480).

This comparison of two classification algorithms and two genotyping techniques indicated that AmpSeq is preferable due to more concordant results, and that the 2/3 algorithm produced most overlap between the two genotyping methods.

## Discussion

This study explored the utility of amplicon deep sequencing for *P. falciparum* genotyping in regulatory clinical trials to assess antimalarial drug efficacy. We showed that down-selected AmpSeq markers together with stringent bioinformatics data analyses produced high quality data, a critical pre-requisite for use in highly regulated PCR-correction of drug failure rates. Genotyping *P. falciparum* clones by AmpSeq offers a number of advantages over other techniques: (i) reproducibility of results is very good as indicated by replicate experiments, sporadic contaminants can be identified and eliminated by performing replicates, (ii) the detection limit of minority clones is clearly defined and not subject to amplification bias owing to different length of the alleles, (iii) multi-clone infections are precisely numbered and unequivocally genotyped, and (iv) the option to quantify each clone and to track its frequency over time gives additional information about the dynamics of resistant clones.

For PCR-correction in regulatory trials, high reproducibility of the typing technique is essential. This was demonstrated by performing three independent replicates of each blood sample. Triplicates were extremely helpful to identify true haplotypes in a very robust manner. This implies additional costs for PCR and sequencing, yet, it is an essential approach to minimize or even eliminate artefacts leading to false haplotypes.

For longitudinal tracking of clones during the clinical trial period over several weeks, the sensitivity of minority clone detection is crucial. A drug resistant clone might be present only as a small proportion of the pre-treatment parasite population in a host, yet, this clone might survive, expand after treatment and even persist despite new incoming clones. For the detection of such a recrudescence, it is of key importance that all haplotypes or size-polymorphic alleles of the recurrent multi-clonal infection are equally well amplified. Major size difference between co-amplified size-polymorphic marker alleles impedes the detectability of clones carrying larger fragment sizes, whereas by AmpSeq such amplification bias has not been observed.

Targeted deep amplicon sequencing in principle is very sensitive to detect minority clones. In our previous work the detection limit of a minority clone was as low as 1:1000 given a sufficient read coverage^15^. In validation experiments for all used AmpSeq markers of the present study, minority clones were detected even up to a ratio of 1:1500 in mixtures of *P. falciparum* strains HB3 and 3D7 (Fig. 1). In the context of the current application in a clinical trial, however, we chose a cut-off ≥1% within-host-haplotype frequency for accepting a haplotype in our study. Despite the high sensitivity for minority clones observed in AmpSeq validation, this was done for two reasons: (i) We aimed to prevent as much as possible signals from persisting gametocytes or residual DNA that might be misclassified as recrudescent parasites, which would lead to overestimation of treatment failure rates^18,19^. Earlier reports showed that hosts after artemisinin-based combination therapy, who were gametocyte-negative by microscopy, still carried sub-microscopic gametocytes detectable by *pfs25* QT-NASBA during trial follow up^20^. In the present clinical trial, it remains unclear, whether gametocytes survived ACT-treatment or derived from recurrent parasitemia. (ii) A further aim was to minimize false haplotypes arising from contamination, as occasionally observed in control samples of mixed *P. falciparum* DNA from *in vitro* culture (Fig. 1, right panel).

Several parameter estimates were compared between AmpSeq and length-polymorphic markers. Multiplicity of infection, i.e. the mean number of concurrent clones per sample, was highly concordant between the established length-polymorphic markers *msp1* and *msp2* and AmpSeq markers (Fig. 3). Also, the genetic diversity was comparable between AmpSeq and length-polymorphic markers in the samples analysed.

The robust detection of minority clones at frequencies as low as 1:100 by AmpSeq represents a key advantage over the established *msp1, msp2* and *glurp* protocol^3^ that has been used routinely for many years. In length-polymorphism-based genotyping, the sensitivity for minority clones greatly depended on the difference in amplicon sizes of mixed strains and a minority clone was only detectable up to a ratio of 1:5 for mixed strains 3D7 / K1 of marker *msp1*^8^. PCR template competition (also known as allelic suppression) acts on PCR fragments that substantially differ in size and are amplified within the same reaction tube. Such biased amplification likely operates as a matter of principle, irrespective of the method applied for distinguishing alleles of the length-polymorphic marker, i.e. sizing by agarose gel electrophoresis, PCR-restriction fragment length-polymorphism and capillary electrophoresis, or by high resolution melt analysis^21–23^, though all these methods differ in their ability to detect minority clones.

Another genotyping technique is based on detection of individual SNPs of a genome-wide distribution and generates so-called molecular barcodes^24^. The major advantage of AmpSeq over molecular barcodes is the potential of AmpSeq to differentiate individual clones within multi-clone infections, even at high multiplicity. On the other hand, major biological limitations in genotyping malaria parasites are not resolvable by neither methods, i.e. daily fluctuations in density and parasite sequestration, hindering longitudinal follow up of parasite clones. These shortfalls of genotyping derive from biological features of the parasite and remain the same irrespective of the typing method used^25^.

The concordance of results among the three genotyped AmpSeq markers was substantially higher than that for length-polymorphic markers. AmpSeq results from markers *ama1-D3*, *cpmp* and *cpp* generally agreed very well and classified 15 paired samples as clear recrudescence and additional 11 pairs as clear NI. Only six paired samples (6/32, 19%) showed discordant results between the three AmpSeq markers. A possible reason for a discrepancy among the three marker results could be that a NI carries by chance the same haplotype as a parasite clone that was present in the pre-treatment sample, but had been successfully cleared. This scenario arising by chance would result in a false classification of a true NI as recrudescence. For length-polymorphic markers, the same problem of false classification applies and largely depends on marker diversity. In addition, an important cause of discrepancies, but only in length-polymorphic markers, is allelic suppression during PCR, which may lead to an allele undetected because of its large size and/or low within-host frequency^8^. This latter problem does not occur with AmpSeq markers. Genotyping by AmpSeq therefore is expected to produce more concordant results than length-polymorphic markers. Indeed, greater concordance between markers was found for AmpSeq, which substantially reduced the number of discrepant results from 34% by length-polymorphic markers to 19% by AmpSeq. This greater concordance by AmpSeq markers represents a major advance over length-polymorphic markers, primarily because the resolution of discording results among the three markers is conditional upon the choice of the WHO versus 2/3 algorithm.

In the past different algorithms were proposed to classify the final PCR-correction outcome in case of discordant results from three markers^1,5,8,9,26^. The 2/3 algorithm was proposed following increased awareness of amplification competition between length- polymorphic fragments, which could lead to loss of minority clones. Recently, Jones and coworkers^27^ have employed pharmacological models to simulate a drug efficacy trial, including therapeutic outcomes and genotyping results based on size-polymorphic markers. In this simulated trial, several molecular correction approaches were tested. The 2/3 algorithm for PCR-correction fitted best with the simulated treatment failure rates.

For AmpSeq markers we compared both these algorithms. By WHO algorithm, all six discordant results were classified as NI, by 2/3 algorithm, four of these were classified as recrudescences based on results of two markers. This shows that the WHO recommended approach tends slightly towards NI, in contrast to the 2/3 algorithm. In the context of clinical trials of antimalarials, it is preferable to be rather more conservative in identifying treatment failure. Following the rationale that overestimation of NI should be avoided as this would prevent the detection of a failing drug, the more stringent classification algorithm, i.e. 2/3, seems justified.

On the other hand, it could be argued that the result of a NI by a single AmpSeq marker constitutes a firm evidence for presence of only new clones in the post-treatment sample because by AmpSeq no suppression of a haplotype owing to size is expected. However, we selected a robust cut-off suitable for a clinical trial, whereby a haplotype needed to reach ≥ 1% of the within-host read frequency. This may increase the probability that a clone can fall below the cut-off in one of the markers, thus leading to a discordant result between three AmpSeq markers. Setting of a read frequency cut-off is required for sequencing data, owing to amplification and sequencing errors. This necessary restriction is traded off against sensitivity to detect minority clones. Owing to this limitation of AmpSeq, we suggest to adopt the 2/3 algorithm also for resolving discordant results from the three AmpSeq markers.

From five available AmpSeq markers, we chose three unlinked single-copy genes, *ama1-D3*, *cpmp* and *cpp*, because these amplicons showed the highest diversity in the pre-treatment samples. The presentation of allelic frequencies in the baseline samples had been recommended in the WHO expert consultation on PCR-correction in clinical drug trials^1^. AmpSeq marker *cpp* was newly developed for this study and revealed 51 haplotypes. This diversity compares to the most polymorphic marker in this study, *cpmp*, with 63 haplotypes. In a previous study conducted in a village in PNG with medium transmission intensity, marker *cpmp* also had shown highest diversity with 27 haplotypes in 37 blood samples^14,15^. The clinical samples of the present study mainly derived from Africa, suggesting that *cpmp* is a suitable marker for various geographic areas.

Marker *csp* in the present study revealed 29 haplotypes in 34 baseline samples, while in the PNG study *csp* had not been sufficiently diverse owing to only three haplotypes identified^15^. Restricted *csp* diversity was also reported from other geographic regions, e.g in SE-Asia^28^ and Peru^29^, whereas *csp* polymorphism was more pronounced in Africa^30^. These findings of geographically varying *csp* diversity highlight the importance of assessing the diversity of molecular markers for each study site, before embarking on a genotyping study.

Another newly developed AmpSeq marker derived from a highly polymorphic region of *msp7*. The selected *msp7* amplicon showed a high value of expected heterozygosity (He = 0.91) in field isolates deposited in the MalariaGEN and was therefore developed as a new AmpSeq marker. Within 34 pre-treatment samples analyzed in this study, however, *msp7* showed the lowest number of haplotypes, with three dominant haplotypes together representing 50% allelic frequency. The latter characteristic poses a problem for recrudescence typing, because, despite a fully acting drug, any new infection would likely carry one of the frequent genotypes. In high transmission areas with high MOI, marker *msp7* could therefore be prone to misclassification and bias towards recrudescence. Because of these high frequencies of the three dominant *msp7* haplotypes we excluded AmpSeq marker *msp7* from our genotyping panel. However, in other geographic regions, marker *msp7* might not be equally restricted and could still be useful.

HaplotypR, our bioinformatic data analysis pipeline, was further developed to support fusion of overlapping paired sequence reads and to allow haplotype calling in triplicates^14,15^. For different study types, the haplotype calling cut-off settings of HaplotypR can be adjusted to permit more or less stringency. For the application of AmpSeq in a clinical trial, a very stringent cut-off of ≥1% within-host haplotype frequency was justified. For other applications of AmpSeq the cut-off may be relaxed, for example, if increased sensitivity for detecting minority clones is required, such as studies of duration of a clonal infection^14^. Recently HaplotypR was validated in comparison to similar bioinformatic pipelines and performed equally well^31^. AmpSeq data analysis critically depends on bioinformatics expertise, an additional requirement for many genotyping laboratories and additional costs. On the other hand, the parallel analysis of multiple AmpSeq markers and the technique’s particular suitability for multiplexing large samples sizes help to lower costs of genotyping. The ease of shipping amplicons globally and ready access to sequencing facilities at academic or commercial resource centres make AmpSeq an option in reach also for laboratories in malaria endemic countries.

### Conclusion

This study showed that AmpSeq genotyping was highly reproducible with robust results that compared well to those from the current standard genotyping technique based on length-polymorphic markers. Performing all AmpSeq experiments in triplicates was an essential precondition for high quality data. We concluded that AmpSeq represents an advance over other techniques owing an unbiased amplification and thus improved sensitivity and reproducibility in the detection of minority clones. In regulatory clinical trials of antimalarial drugs, PCR-correction of failure rates critically depends on the quality of genotyping, we therefore recommend using AmpSeq, as this technique overcomes major shortfalls of other methods.

## Methods

### AmpSeq markers

SNP-rich amplicons were selected from 5 marker genes. Three AmpSeq markers, the genes for circumsporozoite surface protein (*csp*, PF3D7_0304600), apical membrane antigen (*ama1-D3*, PF3D7_1133400), and marker PF3D7_0104100, a conserved plasmodium membrane protein (*cpmp*), had been evaluated previously in *P. falciparum in vitro* culture strains and community samples from Papua New Guinea (PNG)^14,15^. Two additional markers, merozoite surface protein 7 (*msp7*, PF3D7_1335100) and PF3D7_1475800, a conserved plasmodium protein (*cpp)*, were identified using 3411 *P. falciparum* genomes from 23 countries published in the MalariaGEN dataset^16^ according to selection criteria described previously^15^. New markers were selected based on high values for expected heterozygosity (H_e_) in 200 bp windows spanning the SNP-rich regions of the candidate genes. Amplicon sizes were restricted to <450 bp to match length limits of the Illumina MiSeq platform. Diversity of all AmpSeq markers used in this study is presented in Supplementary file, Table S2. Primers were designed manually in conserved regions flanking the polymorphic fragments, yielding a 331 bp fragment for *msp7*, spanning nucleotide positions 575–905, and a 362 bp fragment for *cpp*, spanning nucleotide positions 40–391 (Supplementary file, Table S3). Primers were evaluated *in silico* to exclude formation of primer dimer or secondary structure.

### Archived blood samples from clinical trials of antimalarial drugs (artemisinin-based combination therapy, ACT)

A set of 34 pre- and post-treatment sample pairs, which had been used previously for evaluation of recrudescence typing with length-polymorphic markers *msp2*, *msp1* and *glurp*^8^ was available for AmpSeq analysis to permit direct comparison of methods. The post-treatment sample was collected on the day of recurrent parasitemia following antimalarial treatment with ACT. The median time of recurrence was 21 days (IQR: 14–27 days) post-treatment. These 34 anonymized sample pairs derived from different trial sites across Africa and Asia. Therefore, a description of “overall” endemicity is not possible for this set of samples.

### Ethics statement

Ethical clearance for genotyping of anonymous field samples for the purpose of molecular assay validation was obtained from Ethikkommission Nordwest- und Zentralschweiz (EKNZ Req-2016–00050). All experiments were performed in accordance with relevant guidelines and regulations. Informed consent was obtained from all subjects or, if subjects were under 18, from a parent and/or legal guardian.

### *In vitro* culture stains

Mixtures of *P. falciparum* strains HB3 and 3D7 at six defined ratios were used to determine the limit of detection for minority clones and to validate haplotype calling. Preparation of *P. falciparum* control mixtures was described previously^8^.

### DNA Extraction

For all clinical trials samples, total DNA was extracted from dried blood spots (DBS) on Whatman 3MM filter paper. Three disks of 3 mm diameter were punched from a dried blood spot, washed in 500 µL dH2O and boiled in 35 µL dH2O for 30 min at 98 °C with brief vortexing every 30 sec to elute gDNA. Extracted DNA was stored at 4 °C.

### Primary and nested PCR

To enrich target sequences from extracted total DNA, a primary PCR was performed as described previously with the following modifications^15^. Primary PCRs were prepared in duplex *(ama1-D3*/*cpmp*) and triplex (*csp*/*cpp*/*msp7*) reactions in a final volume of 15 µL including 3 µL DNA as template, 250 nm of each primer pair and 7.5 µL KAPA HiFi HotStart Ready Mix (Roche). Primer sequences and thermo profiles are provided in the Supplementary file, Tables S3–S6.

Individual nested PCRs (nPCR) were performed for all 5 markers to ensure high yields of product (Supplementary file, Table S6). This step added a 5‘-linker for later adapter addition to the primary PCR (pPCR) product. nPCR reactions were prepared in a final volume of 15 µL including 1 µL of pPCR product, 250 nm nPCR primer pairs and 7.5 µL KAPA HiFi HotStart Ready Mix (Roche). To confirm successful amplification nPCR products were run on a 2% agarose gel.

### Normalisation of nPCR products

nPCR products were normalised using a SequalPrep^TM^ Normalisation plate (Thermo Fisher Scientific) according to the manufacturer’s instructions. Briefly, 8 µL nPCR products and 10 µL binding buffer were applied to the normalisation plate. After 1 h incubation, nPCR products were eluted in 15 µL elution buffer yielding a final DNA concentration of 1–2 ng/ µL. 5 µL of normalised nPCR products (*ama1-D3*/*cpmp* or *csp*/*cpp*/*msp7*) were pooled and used as template for adapter PCR.

### Multiplexed adapter-addition PCR

Individual samples were barcoded with a unique forward and reverse sequence index by performing a third round of PCR, which was performed as duplex *(ama1-D3*/*cpmp*) and triplex (*csp*/*cpp*/*msp7*) reaction in a final volume of 15 µL including 1 µL of template DNA, 833 nm adapter primer pairs and 7.5 µL KAPA HiFi HotStart Ready Mix (Roche). PCR products were run on a 2% agarose gel.

### Amplicon library preparation

Twenty samples of either the *ama1-D3*/*cpmp* or the *csp*/*cpp*/*msp7* multiplexed adapter-addition PCR reaction were combined to pools of equal molarity. These pools were purified with 0.6 volumes of NucleoMag beads (Macherey-Nagel) and quantified by Qubit fluorometer (Thermo Fisher Scientific). In a final step, each pool was diluted to 10 nM and combined to a final sequencing library.

### Illumina MiSeq sequencing

Prior to deep sequencing, correct amplicon sizes in library pools were confirmed by Agilent 4200 TapeStation System. Final amplicon libraries were sequenced in two runs on an Illumina MiSeq platform in paired-end mode using the MiSeq reagent kit v3 (600 cycles; 2 × 300 bp) including 10% spike-in of Enterobacteria phage *phiX* control v3 (Illumina).

### Sequence read analysis and haplotype calling

Deep sequencing reads were analysed using bioinformatic pipeline HaplotypR version 0.3 (https://github.com/lerch-a/HaplotypR/releases/tag/v0.3)^14,15^. Sequencing reads were demultiplexed by sample and by amplicon. Overlapping sequence of paired reads were merged using software vsearch (parameters:–fastq_mergepairs–fastq_truncqual 1–fastq_maxns 0)^32^ and clustered using software swarm^33^. Samples that showed a read coverage <50 reads per sample were excluded from the analysis. SNP calling required a minimum 50% mismatch rate and a minimum occurrence in 2 independent samples^15^. The term haplotype in our work denotes “unique sequence variant of an entire amplicon”. Haplotypes resulting from insertion and deletion (indels), chimeric or singleton reads were removed and thus excluded from further analysis. Haplotype calling followed stringent cut-off criteria and required: (i) a minimum read coverage of 10 reads per haplotype and sample, (ii) a within-host haplotype frequency ≥1%, and (iii) a minimum occurrence of the haplotype in 2 out of the 3 replicates. Haplotypes occurring only once among triplicates of a sample were excluded. Within-host-haplotype frequency was derived from the number of reads per haplotype per amplicon over the sum of all reads per amplicon in a sample. Final haplotype sequences obtained in this study were deposited in GenBank under the accession numbers MK975258 - MK975308 (*csp*), MK975309 - MK975380 (*ama1-D3*), MK981569-MK981614 (*msp7*), MN010910 - MN010993 (PF3D7_0104100), MN010994 - MN011066 (PF3D7_1475800).

### Classification of samples into new infection and recrudescence

The final PCR-correction were established in two steps. In the first step, the genotyping result, i.e. new infection (NI) or recrudescence (R) was determined for each of the three markers *ama1-D3*, *cpmp* and *cpp* according to the following criteria: Classification of recrudescence (R) required that at least one of the haplotypes occurred in both, the pre- and post-treatment sample, with a minimum haplotype frequency of 1% in at least two of the three independent replicates performed. A new infection (NI) was defined by the occurrence of only new haplotypes in the post-treatment sample with a minimum haplotype frequency of 1%. This definition corresponds to that one established in 2007 by experts in the field during a WHO/MMV-convened meeting on genotyping methods for clinical trials on antimalarial drug efficacy^1^.

In the second step, the final outcome for each sample was derived from all three markers, *ama1-D3*, *cpmp* and *cpp*. In case any of the markers fell below the minimum read criterion, marker *csp* was used to complement a full set of three markers. In case all markers agreed, this concordant result was directly reported as final PCR-corrected treatment outcome. In case R and NI classification of individual markers disagreed, two different approaches (here called algorithms) were applied to resolve the discordancy: (i) The WHO/MMV recommended approach^1^, where a recurrent parasitemia is classified in the overall outcome as NI, if at least one of the three markers had given a NI result. (ii) The alternative, newer approach termed “2/3 algorithm”^8^. This algorithm uses the consensus of two markers as final PCR-correction outcome. We report the PCR-correction results for both algorithms, and discuss their respective benefits.

## Supplementary information

Supplementary File

## Data Availability

The datasets generated and analysed in the course of this study are available in NCBI Sequence Read Archive repository under accession numbers SRR8608898 and SRR8608897. Haplotype sequences were deposited in GenBank under the accession numbers MK975258 - MK975308 (*csp*), MK975309 - MK975380 (*ama1-D3*), MK981569-MK981614 (*msp7*), MN010910 - MN010993 (PF3D7_0104100), MN010994 - MN011066 (PF3D7_1475800). The source code for software HaplotypR version 0.3.1 is available at https://github.com/lerch-a/HaplotypR/releases/tag/v0.3.1.
