# Supplementary material for: Amplicon deep sequencing improves Plasmodium falciparum genotyping in clinical trials of antimalarial drugs
Source: Sci Rep. 2019 Nov 28;9:17790. doi: 10.1038/s41598-019-54203-0 (PMC6883076; doi:10.1038/s41598-019-54203-0)
Supplement: Supplementary file 1 — Supplementary File [file 41598_2019_54203_MOESM1_ESM.docx]

**Amplicon deep sequencing improves *Plasmodium falciparum* genotyping in clinical trials of antimalarial drugs**

Authors: Maria Gruenberg, Anita Lerch, Hans-Peter Beck, Ingrid Felger

**SUPPLEMENTARY FILE**

**Table S1.** Median sequence read coverage per sample and marker

|  | **Sequence reads/sample**  **Median [IQR]** | |
| --- | --- | --- |
| **Marker** | **Day 0** | **Day X** |
| *ama1-D3* | 17224  [1756-24469] | 11662  [5842-18103] |
| *cpmp* | 11109  [784-19786] | 8208  [2747-24804] |
| *cpp* | 17712  [1679-23844] | 16488  [6934-25012] |
| *csp* | 14712  [1306-22398] | 14684  [8052-21546] |
| *msp7* | 14573  [839-19204] | 10191  [2427-16805] |

Table S2. Number of haplotypes and expected heterozygocity (H_e_) of 5 AmpSeq amplicons in 3411 *P. falciparum* isolates from MalariaGEN database

| **Marker** | **PlasmoDB Annotation** | **Haplotypes** | **H_e_** |
| --- | --- | --- | --- |
| *ama1-D3* (PF3D7_1133400) | Apical membrane antigen1 | 79 | 0.93 |
| *cpmp*  (PF3D7_0104100) | Conserved Plasmodium protein | 82 | 0.93 |
| *csp*  (PF3D7_0304600) | Circumsporozoite protein | 77 | 0.85 |
| *cpp*  (PF3D7_1475800) | Conserved Plasmodium protein | 77 | 0.92 |
| *msp7*  (PF3D7_1335100) | Merozoite protein7 | 67 | 0.91 |

**Table S3.** PCR Primer sequences for AmpSeq library preparation [1, 2]

| **Primer for primary PCR** | |
| --- | --- |
| ama1-D3_prim_fw | GTTTAATTAACAATTCATCATAC |
| ama1-D3_prim_rv | GTGTTGTATGTGATGCTC |
| cpmp_prim_fw | CGATACAGGACATATAGA |
| cpmp_prim_rv | TTCAATAACATTTACTAGG |
| cpp_prim_fw | TGTCTGAACCAAATTCAA |
| cpp_prim_rv | GAATTTGTCACATTTGATGA |
| csp_prim_fw | ATCAAGGTAATGGACAAG |
| csp_prim_rv | ACTCAAACTAAGATGTGTTC |
| msp7_prim_fw | GTATTATCAAAGGTAAAGGCA |
| msp7_prim_rv | TTGCATAACTATAAACACCAT |
|  | |
| **Primer for nested PCR** | |
| ama1-D3_fw_linker | **GTGACCTATGAACTCAGGAGTC**TACTACTGCTTTGTCCCATC |
| ama1-D3_rv_linker | **CTGAGACTTGCACATCGCAGC**TCAGGATCTAACATTTCATC |
| cpmp_fw_linker | **GTGACCTATGAACTCAGGAGTC**CATAAGTCATTAAAATTTATGGAT |
| cpmp_rv_linker | **CTGAGACTTGCACATCGCAGC**CGTTACTATCAAGATCGTTAATATC |
| cpp_fw_linker | **GTGACCTATGAACTCAGGAGTC**CAAGTTCACTTTTGGGAAATG |
| cpp_rv_linker | **CTGAGACTTGCACATCGCAGC**ATTACTACCTTTCAGCATATCCGA |
| csp_fw_linker | **GTGACCTATGAACTCAGGAGTC**AAATGACCCAAACCGAAATGT |
| csp_rv_linker | **CTGAGACTTGCACATCGCAGC**GGAACAAGAAGGATAATACCA |
| msp7_fw_linker | **GTGACCTATGAACTCAGGAGTC**ATGAACAAGAGATATCAACACA |
| msp7_rv_linker | **CTGAGACTTGCACATCGCAGC**TTAAATTGTTCATGGTATTCCTTA |

References

1. Lerch A, Koepfli C, Hofmann NE, Messerli C, Wilcox S, Kattenberg JH, et al. Development of amplicon deep sequencing markers and data analysis pipeline for genotyping multi-clonal malaria infections. BMC Genomics. 2017;18(1):864. doi: 10.1186/s12864-017-4260-y. PubMed PMID: 29132317; PubMed Central PMCID: PMCPMC5682641.

2. Lerch A, Koepfli C, Hofmann NE, Kattenberg JH, Rosanas-Urgell A, Betuela I, et al. Longitudinal tracking and quantification of individual Plasmodium falciparum clones in complex infections. Sci Rep. 2019;9(1):3333. doi: 10.1038/s41598-019-39656-7. PubMed PMID: 30833657; PubMed Central PMCID: PMCPMC6399284.

**Table S4.** PCR Primer sequences for AmpSeq library preparation

| **Primer for Adapter PCR (XXXXXXXX=barcode)** | | | |
| --- | --- | --- | --- |
| Forward | AATGATACGGCGACCACCGAGATCTACACTCTTTCCCTACACGACGCTCTTCCGATCT**XXXXXXXX**GTGACCTATGAACTCAGGAGTC | | |
| Reverse | CAAGCAGAAGACGGCATACGAGATCGGTCTCGGCATTCCTGCTGAACCGCTCTTCCGATCT**XXXXXXXX**CTGAGACTTGCACATCGCAGC | | |
|  | | | |
| **Forward barcode** | | **Reverse barcode** | |
| Fw_1 | TAGATCGC | Rv_1 | TAAGGCGA |
| Fw_2 | CTCTCTAT | Rv_2 | CGTACTAG |
| Fw_3 | TATCCTCT | Rv_3 | AGGCAGAA |
| Fw_4 | AGAGTAGA | Rv_4 | TCCTGAGC |
| Fw_5 | GTAAGGAG | Rv_5 | GGACTCCT |
| Fw_6 | ACTGCATA | Rv_6 | TAGGCATG |
| Fw_7 | AAGGAGTA | Rv_7 | CTCTCTAC |
| Fw_8 | CTAAGCCT | Rv_8 | CAGAGAGG |
| Fw_9 | CCGAAGTA | Rv_9 | GCTACGCT |
| Fw_10 | GAGCTGAA | Rv_10 | CGAGGCTG |
| Fw_11 | GCGAGTAA | Rv_11 | AAGAGGCA |
| Fw_12 | TGAAGAGA | Rv_12 | GTAGAGGA |
| Fw_13 | TGGTGGTA | Rv_17 | CGCATACA |
| Fw_14 | TTCACGCA |  |  |

**Table S5:** Thermo profiles of primary, nested and adapter PCR

| **Primary PCR** | |  | |  |
| --- | --- | --- | --- | --- |
| **Programme** | **Temperature** | **Time** | | **Cycles** |
| Initial denaturation | 95°C | 3min | |  |
| Denaturation | 98°C | 20sec | |  |
| Annealing | 54^1^°C | 15sec | | 20 |
| Elongation | 72°C | 45sec | |  |
| Final elongation | 72°C | 2min | |  |
|  |  |  | |  |
| **Nested PCR** | |  | |  |
| **Programme** | **Temperature** | **Time** | | **Cycles** |
| Initial denaturation | 95°C | 3min | |  |
| Denaturation | 98°C | 20sec | |  |
| Annealing | 55°C | 15sec | | 10 |
| Elongation | 72°C | 45sec | |  |
|  |  |  | |  |
| Denaturation | 98°C | 20sec | |  |
| Annealing | 62°C | 15sec | | 10 |
| Elongation | 72°C | 45sec | |  |
| Final elongation | 72°C | 1.30min | |  |
| **Adapter PCR** |  |  |  |  |
| **Programme** | **Temperature** | **Time** | | **Cycles** |
| Initial denaturation | 95°C | 3min | |  |
| Denaturation | 98°C | 20sec | |  |
| Annealing | 58*°C | 30sec | | 10* |
| Elongation | 72°C | 45sec | |  |
| Final elongation | 72°C | 2min | |  |

^1^ for amplicon *msp7*/*cpp*/*csp* only; for amplicon *cpmp*/*ama1-D3* 52°C were applied

**Table S6.** PCR reaction mix of primary, nested and adapter PCR

|  |  |  |  |
| --- | --- | --- | --- |
| **Primary PCR** |  |  |  |
| **Reagents** | **Stock concentration** | **V (µL)** | **Final concentration** |
| KAPA HiFi HotStart ReadyMix | 2x | 7.5 | 1x |
| Primer pair 1 (fw/rv) | 10µM | 0.375 | 250nM |
| Primer pair 2 (fw/rv) | 10µM | 0.375 | 250nm |
| Primer pair 3 (fw/rv) | 10µM | 0.375 | 250nM |
| H2O |  | 3.375 |  |
| Template |  | 3 |  |
| **Total** |  | **15** |  |
|  |  |  |  |
| **Nested PCR** |  |  |  |
| **Reagents** | **Stock concentration** | **V (µL)** | **Final concentration** |
| KAPA HiFi HotStart ReadyMix | 2x | 7.5 | 1x |
| Primer pair (fw/rv) | 10µM | 1.25 | 250nM |
| H2O |  | 5.25 |  |
| Template | pPCR | 1 |  |
| Total |  | **15** |  |
|  |  |  |  |
| **Adapter PCR** |  |  |  |
| **Reagents** | **Stock concentration** | **V (µL)** | **Final concentration** |
| KAPA HiFi HotStart ReadyMix | 2x | 7.5 | 1x |
| Primer pair fw | 10µM | 1.25 | 833nM |
| Primer pair rv | 10µM | 1.25 | 833nM |
| H2O |  | 4 |  |
| Template | nPCR | 1 |  |
| Total |  | **15** |  |

**Note: Singleplex versus multiplex PCR**

Different PCR conditions were tested prior to analysing samples of a clinical trial. With the aim to reduce the work load during amplicon library construction, we evaluated multiplex PCR for the equally sized amplicons (*ama1-D3*/*cpmp*) and (*cpp*/*csp*/*msp7*). Multiplexing of the primers for the two markers *ama1-D3*/*cpmp* and the three markers *cpp*/*csp*/*msp7* worked well for the first (target enrichment) round of PCR. Whereas in the second round of PCR, multiplexing resulted in reduced PCR efficiency and therefore lower yield of PCR product. Lower yields are likely attributed to primer dimer formation of long overhang primers. To ensure optimal amplification of all 5 markers, only singleplex reactions were run in the second round of PCR.

**Figure S1.** **PCR-correction outcomes of 28 Day 0/Day X sample pairs**. Within-host haplotype frequencies are shown for AmpSeq markers *ama1-D3*, *cpmp*, and *cpp*, quantified by three replicates (Rep1 to Rep3) for each of the Day 0 and Day X samples. Different haplotypes of a marker are represented by different shapes and colours.

**Figure S2.** **PCR-correction outcomes of three Day 0/Day X sample pairs after replacement of marker *cpmp* by backup marker *csp*.** Within-host haplotype frequencies are shown for AmpSeq markers *ama1-D3*, *cpp*, and *csp* (because marker *cpmp* had failed to amplify). Haplotypes were quantified by three replicates (Rep1 to Rep3) for each of the Day 0 and Day X samples. Different haplotypes of a marker are represented by different shapes and colours.
